# Investigation and management of moderate to severe inpatient hyponatraemia in an Australian tertiary hospital

**DOI:** 10.1186/s12902-018-0320-9

**Published:** 2018-12-06

**Authors:** Kathryn Berkman, Kate Haigh, Ling Li, Jack Lockett, Goce Dimeski, Anthony Russell, Warrick J. Inder

**Affiliations:** 1 Department of Diabetes and Endocrinology, Princess Alexandra Hospital, 199 Ipswich Road, Woolloongabba, Brisbane, QLD 4102 Australia; 2 Department of Chemical Pathology, Pathology Queensland, Brisbane, Queensland Australia; 3 Faculty of Medicine, The University of Queensland, Brisbane, Queensland Australia

**Keywords:** Hyponatraemia, SIADH, Fluid restriction, Urea

## Abstract

**Background:**

Hyponatraemia is the most common electrolyte disturbance amongst hospitalised patients. Both American and European guidelines recommend fluid restriction as first line treatment for SIADH, however differ on second line recommendations. The objective of this study was to examine investigation and management of hyponatraemia in hospitalised patients in an Australian tertiary hospital.

**Methods:**

A retrospective audit was conducted of electronic medical records and laboratory data of inpatients with serum sodium (Na) ≤125 mmol/L, admitted over a 3 month period to the Princess Alexandra Hospital, Brisbane, Australia. The main outcomes measured included: demographic characteristics, investigations, accuracy of diagnosis, management strategy, change in Na and patient outcomes.

**Results:**

The working clinical diagnosis was considered accurate in only 37.5% of cases. Urine Na and osmolality were requested in 72 of 152 patients (47.4%) and in 43 of 70 euvolaemic patients (61.4%). Thyroid function tests (67.1%) and morning cortisol (45.7%) were underutilized in the euvolaemic group. In the SIADH cohort, fluid restriction resulted in a median (IQR) 7.5 mmol/L (4–10.5) increase in Na after 3 days; no treatment resulted in a median 0 mmol/L (− 0.5–1.5) change. Oral urea was utilized in 5 SIADH patients where Na failed to increase with fluid restriction alone. This resulted in a median 10.5 mmol/L (3.5–13) increase in Na from baseline to day 3. There were no cases of osmotic demyelination. The median length of stay was 8 days (4–18.5). Mortality was 11.2% (17 patients). There was a weak but significant correlation between nadir serum Na and mortality (*R* = 0.18, *P* = 0.031).

**Conclusion:**

Inpatient hyponatraemia is often inadequately investigated, causing errors in diagnosis. Treatment is heterogeneous and often incorrect. In cases with hyponatraemia refractory to fluid restriction, oral urea presents an effective alternative treatment.

## Background

Hyponatraemia is defined by a serum sodium (Na) concentration <135 mmol/L [1]. It is the most common electrolyte disturbance amongst hospitalised patients at a rate of 15–30% [1–3]. A prospective cohort study of 98,411 hospitalised patients demonstrated that even mild hyponatraemia (Na 130–134 mmol/L) is associated with increased mortality in hospital (OR 1.37), at 1 year (OR 1.35) and at 5 years post discharge (OR 1.24) [4].

Traditionally, hyponatraemia is classified clinically by volume status into hypovolaemic, euvolaemic and hypervolaemic hyponatraemia [1, 5]. The most common cause of euvolaemic hyponatraemia is the syndrome of inappropriate antidiuretic hormone (SIADH) [3, 6], which is characterized by dysregulated antidiuretic hormone (ADH) secretion and water retention, despite low serum Na and osmolality [5–7]. Diagnostic criteria include: euvolaemic state, normal thyroid hormone and cortisol levels, serum Na <135 mmol/L, reduced serum osmolality <275 mOsm/kg, urine osmolality >100 mOsm/Kg and urine Na >30 mmol/L [2].

Hyponatraemia can present a diagnostic challenge in patients with cancer [8]. SIADH is the most common aetiology in this setting and may be secondary to lung or brain neoplasm, paraneoplastic syndromes or as a result of chemotherapy agents, NSAIDs or opioids [8]. Other aetiologies to be considered include excessive fluid administration and dehydration [8].

Identifying the correct aetiology of hyponatraemia requires a thorough history, physical examination and consideration of relevant biochemical results. Serum electrolytes (including Na, glucose and urea), serum osmolality, with paired urine Na and osmolality are all necessary to establish the correct diagnosis [1, 2]. In the case of euvolaemic hyponatraemia, adrenal insufficiency and hypothyroidism should also be excluded [2], although in practice hypothyroidism is rarely a cause of hyponatraemia [1].

The treatment of hyponatraemia depends on both the underlying aetiology and the timeframe in which it develops [1, 2, 8]. The recommendation by both the American expert panel and European clinical practice guidelines for first line treatment of chronic euvolaemic hyponatraemia, with either mild or absent symptoms is fluid restriction; however, the paucity of supportive data for this is acknowledged [1, 2]. There is no clear guidance regarding the volume to which fluid intake should be restricted. Some groups advocate the use of the urine/plasma electrolyte ratio as a predictor of non-response to fluid restriction due to negative free water clearance if >1 [9, 10], while it has been previously reported that a urine osmolality of >400 mOsm/kg correlated with failure of fluid restriction [11].

The underlying aetiology and outcomes of hyponatraemia have been examined previously in Australian studies [12, 13], however the investigation, accuracy of diagnosis and detailed management practices have never been described in the Australian setting. The aim of this study is to examine the aetiology, investigation and treatment of moderate to severe hyponatraemia in an Australian tertiary hospital.

## Methods

Case finding was undertaken using the pathology laboratory program AUSLAB at the Princess Alexandra Hospital, an adult tertiary referral centre with >100,000 admissions per year. Patients who were admitted from the 1st of March to 31st of May 2016, and had moderate to severe hyponatraemia, defined as a serum Na ≤125 mmol/L at any point during an admission were identified. This degree of hyponatraemia was chosen as clinically significant, because serum Na >125 mmol/L has been correlated with an absence of symptoms in prior studies [14, 15]. Medical records were reviewed by three investigators (KB, KH, LL). Exclusion criteria included age <18 years, pregnancy and pseudohyponatraemia secondary to hyperglycaemia or hyperlipidaemia.

Parameters recorded included: patient demographics, medications, serum and urine electrolytes, thyroid and adrenal function. Duration of hyponatraemia was determined from the available blood test results and from the clinical record. If the duration was unable to determined it was classified as unknown. Volume status was assessed by treating team documentation (noted as hypo-, eu- or hypervolaemic) or by documentation of clinical features such as capillary refill, pulse rate, blood pressure (including postural blood pressure), jugular venous pressure, mucous membranes and presence of pulmonary or peripheral oedema. Symptoms of hyponatraemia such as headache, nausea and vomiting, confusion, seizures and coma were noted [5, 6]. The treating team’s working diagnosis (if documented) was also recorded. Patients were analysed and re-classified into likely underlying cause of hyponatraemia if this could be determined from the documentation of fluid status as well as available investigation results. A diagnosis of SIADH was considered probable if the criteria presented by Spasovski et al. were met [2]. A diagnosis of non-renal salt depletion was deemed likely if the urinary Na was <30 mmol/L in the setting of hypovolaemia. A diagnosis of fluid overload was deemed likely if the urinary Na was <30 mmol/L in conjunction with clinical features of hypervolaemia such as oedema, ascites or pleural effusions [2]. Patient diuretic use was assessed to be a significant contribution to hyponatraemia if the urine Na was ≥30 mmol/L [2]. Primary polydipsia causing relative water excess was diagnosed if the urine osmolality was <100 mOsm/kg [2]. Information was also collected regarding management, change in serum Na (Δ Na) and outcomes.

The data were analysed using Statistical Package for the Social Sciences (SPSS), Version 22. Continuous variables were tested for normality using the Shapiro-Wilk test. Based on the outcome, nonparametric tests were carried out and data presented as median and interquartile range (IQR). For correlations between categorical variables chi square testing was performed. Kruskal-Wallis analysis of variance was performed to compare between group differences. Spearman’s rank-order correlation was utilized to determine relationships between continuous variables and logistic regression to determine relationships between mortality and dependent variables. *P* <0.05 was considered statistically significant.

## Results

There was a total of 9774 admissions (excluding day admissions) over the three-month period. One hundred fifty-two inpatients (1.6% of admissions) were identified with a serum Na ≤125 mmol/L with a median age of 66 years (IQR: 54–75 years). Patients were predominantly admitted under surgical units (41 of 152 patients (27%), General Medicine (38 patients (25%)); followed by Cardiology (16 patients (10.5%)), Nephrology (13 patients (8.6%)), Gastroenterology (13 patients (8.6%)) and Respiratory (7 patients (4.6%)). Table 1 summarizes the patient demographics, comorbid conditions, contributory medications, symptoms and estimated duration of hyponatraemia.

**Table 1:** Patient demographics

	Number	Percent
Gender		
Males	98	64.5%
Females	54	35.5%
Admission Diagnosis		
Infection	28	18.4%
Hyponatraemia	22	14.5%
Elective Admission	15	9.9%
Cancer or cancer related complication	15	9.9%
Chronic liver disease	9	5.9%
Acute Coronary Syndrome or Arrhythmia	9	5.9%
Fall	8	5.3%
Fracture	7	4.6%
Congestive heart failure	6	3.9%
Intracerebral haemorrhage / CVA	6	3.9%
Psychiatric condition	3	2.0%
Other	24	15.8%
Comorbid conditions		
Chronic kidney disease	30	19.7%
Congestive heart failure	22	14.5%
Chronic liver disease	22	14.5%
Contributing Medication on admission		
Angiotensin II Receptor blocker	25	16.4%
ACE inhibitor	24	15.8%
Antidepressant	18	11.8%
Anticonvulsant	13	8.6%
Antipsychotic	11	7.2%
Pregabalin	6	3.9%
Diuretic Use on Admission		
Frusemide	31	20.4%
Spironolactone	15	9.9%
Thiazide	9	5.9%
Indapamide	1	0.7%
Duration of Hyponatraemia		
Chronic (> 48 h duration)	77	50.7%
Acute	10	6.6%
Unknown	65	42.8%
Symptoms of Hyponatraemia		
None	106	69.7%
Nausea/vomiting	21	13.8%
Lethargy	11	7.2%
Confusion	9	5.9%
Decreased level of consciousness	5	3.3%
Seizure	2	1.3%

In 106 patients (69.7%), there were no documented symptoms associated with hyponatraemia. The most prevalent symptom recorded was nausea and/or vomiting in 21/152 patients (13.8%). There was a higher incidence of reported symptoms in patients with more severe hyponatraemia, with symptoms reported in 20/30 patients with a Na level <120 mmol/L, compared with only 26/122 patients of patients with a serum Na 120–125 mmol/L, chi square analysis *P* <0.01. The median (IQR) nadir serum Na in the symptomatic group was 121 mmol/L (115–124) compared with 124 mmol/L (122–125) in the asymptomatic group, Mann Whitney U - *P* <0.01).

### Investigation and diagnosis

In the entire cohort, urine Na and osmolality were performed in 72/152 patients (47.4%). These tests were more frequently ordered in patients who were assessed to be hypovolaemic or euvolaemic. Similarly, thyroid function tests and morning cortisol were underutilized in the euvolaemic group. Table 2 summarizes the investigations ordered by the treating team. Table 3 presents the median biochemical results according to diagnosis.

**Table 2:** Proportion of patients with moderate to severe hyponatraemia undergoing specific investigations

Fluid balance	Whole group	Hypovolaemic	Euvolaemic	Hypervolaemic	Fluid status not documented
N (%)	152 (100)	25 (16.4)	70 (46.1)	35 (23)	22 (14.5)
Urine Na	78 (51.3)	16 (64)	47 (67.1)	12 (34.3)	3 (14.3)
Urine Osmolality	73 (48)	14 (56)	43 (61.4)	12 (34.3)	4 (19)
Thyroid Function tests	92 (60.5)	17 (68)	47 (67.1)	19 (54.3)	9 (42.9)
Morning Cortisol	53 (35)	8 (32)	32 (45.7)	10 (28.6)	3 (14.3)

Urine Na and osmolality, thyroid function tests and morning cortisol are displayed as number of patients who had the test performed (%)

**Table 3:** Biochemistry results. Initial Na, nadir Na, initial osmolality, urea and creatinine are presented as median (IQR)

Fluid balance	Whole group	Hypovolaemic	Euvolaemic	Hypervolaemic	Fluid status not documented
N (%)	152 (100)	25 (16.4)	70 (46.1)	35 (23)	22 (14.5)
Initial serum Na (mmol/L)	124 (121–125)	123 (120.5–125)	123.5 (119.75–125)	124 (121–125)	124 (121.75–125)
Nadir serum Na (mmol/L)	123 (121–125)	123 (120.5–125)	123 (119–125)	123 (119–125)	124 (121.8–125)
Initial serum Osm (mOsm/kg)	261 (254–268)	264 (255.5–273.8)	259 (252–263)	264 (255–274)	257.5 (255.3–266.8)
Initial Urea (mmol/L)	5.6 (3.7–10.1)	7.2 (5.23–12.2)	4.4 (3.1–6.2)	9.1 (5–16.2)	4.4 (3.2–10.3)
Initial Creatinine (μmol/L)	73.5 (56.3–227)	89 (63.5–152)	67.5 (51.5–90.5)	112 (68–244)	64.5 (52.5–108.25)
Urine Na (mmol/L)	35 (22–70.75)	23.5 (15.75–27.5)	45 (31.5–74)	25.5 (21.25–71)	32.5 (27.25–37.75)
Urine Osm (mOsm/kg)	310 (205.5–426)	233.5 (204–357.25)	330.5 (234.25–500.75)	276.5 (209–389.75)	223 (116.75–280.25)

**Fig. 1:**
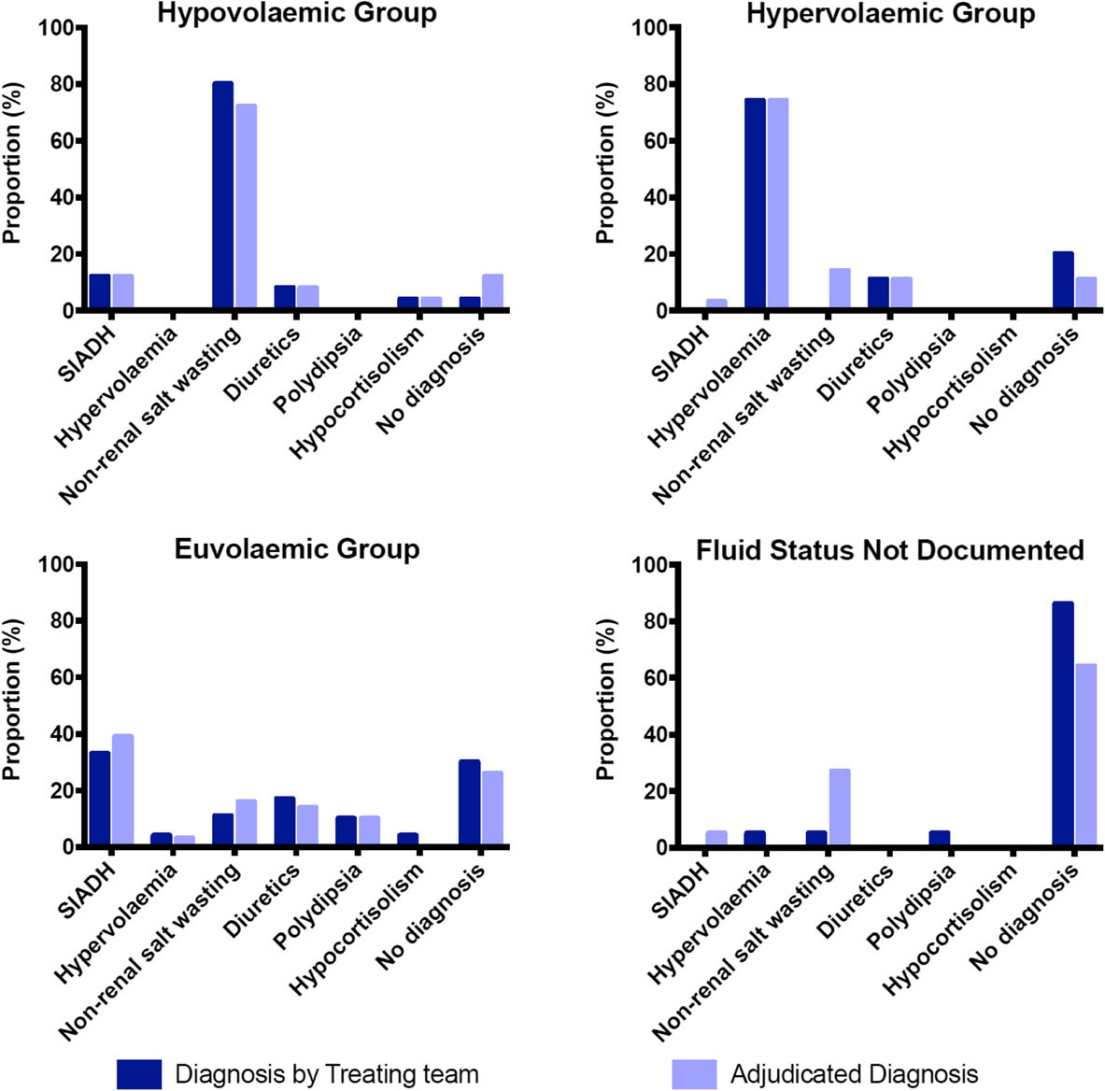
Working diagnosis by treating team and retrospective adjudicated diagnosis after review

There was discordance in the treating team’s working diagnosis compared with the adjudicated diagnosis. In particular, there was a reduction in the number of patients who had no diagnosis from 31.6% of patients to 25.7% after review by investigators. In 34 cases (22.4%), the working clinical diagnosis was inconsistent with the available clinical and biochemical information. Figure 1 details the working diagnosis by treating team and retrospective adjudicated diagnosis after review.

### Management

Daily fluid balance was fully documented in 49/152 patients (32.2%), was documented incompletely in 20 patients (13.2%) and was not documented in 83 patients (54.6%).

Diuretics were ceased in 21/152 patients (13.8%), most of whom were presumed to be hyponatraemic secondary to diuretics ± other diagnoses, in all but 1 patient (no diagnosis recorded). An ACE inhibitor or ARB was ceased in 3 patients, 2 of whom no diagnosis was recorded and 1 of whom was thought to have hyponatraemia secondary to diuretics.

For the patients diagnosed with SIADH by the treating team, fluid restriction was the most common treatment, used in 17/24 patients. Table 4 summarises the full treatment details. In this group, 21 were judged as correctly diagnosed with SIADH, whereas 1 patient had hyponatraemia secondary to non-renal salt wasting and would have received inappropriate treatment.

**Table 4:** Treatment of hyponatraemia according to treating team’s working diagnosis. Patients with multiple diagnoses have been omitted. Patients with multiple treatment modalities have been counted under each treatment

	SIADH	Hypervolaemia	Non renal salt wasting	No diagnosis	Polydipsia
N (%)	24 (15.8)	25 (16.4)	29 (19.1)	48 (31.6)	8 (5.3)
Fluid Restriction	17 (70.8)	17 (68)	1 (4)	6 (12.5)	4 (50)
0.9% saline	1 (4.2)	1 (4)	23 (92)	7 (14.6)	2 (25)
3% saline	2 (8.3)	0 (0)	0 (0)	1 (2.1)	3 (37.5)
Fludrocortisone	1 (4)	0 (0)	0 (0)	0 (0)	0 (0)
No Treatment	2 (8)	2 (8)	1 (4)	32 (66.7)	1 (12.5)
0.9% saline ceased	1 (4)	0 (0)	0 (0)	0 (0)	0 (0)
Urea	5 (20)	0 (0)	0 (0)	0 (0)	0 (0)
Frusemide	0 (0)	9 (36)	0 (0)	1 (2.1)	0 (0)
Dialysis	0 (0)	1 (4)	0 (0)	0 (0)	0 (0)
Diuretics ceased	0 (0)	0 (0)	0 (0)	2 (4.2)	0 (0)
ACE inhibitor or Angiotensin II receptor blocker ceased	0 (0)	0 (0)	0 (0)	2 (4.2)	0 (0)

**Fig. 2:**
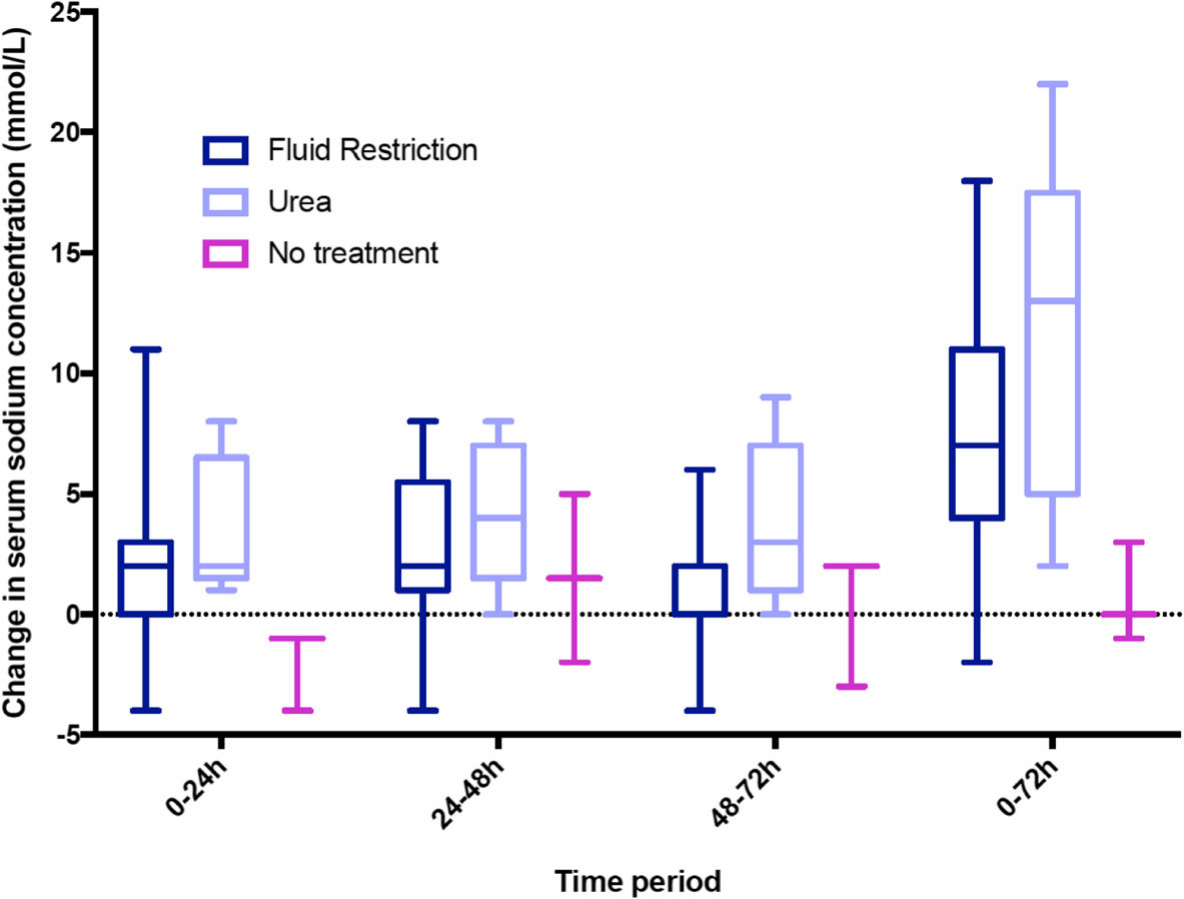
Median change in serum sodium (Δ Na) in adjudicated SIADH group between 0 and 72 h

Amongst the patients who were likely to have SIADH based on retrospective analysis, 16/27 patients were treated with fluid restriction, 1 with 0.9% saline, 5 with oral urea and 5 received no treatment. The vaptan class of drugs was not available for use at our institution during the study period. The time course of Δ Na comparing fluid restriction, urea and no treatment is presented in Fig. 2. The initial fluid restriction volume ranged from 275 to 1500 mL with a median of 1000 mL (625–1375 mL). Neither the volume of fluid restriction nor the median Δ Na after 72 h differed significantly between patients with a urine Osm greater or less than 400 mOsm/kg. There was no correlation between fluid restriction volume and change in serum Na from day 1 to day 2 (*R* = 0.057, *P* = 0.8).

Urea was prescribed in 5 patients (dose range 15–90 g/ day), who had either failed to demonstrate a rise in serum Na whilst fluid restricted or were unable to be fluid restricted. All 5 were diagnosed with SIADH by the treating team and met diagnostic criteria for SIADH on retrospective review. Minor adverse effects were reported in 3 of the 5 patients. These included unpleasant taste (1 patient), nausea (1 patient) and gastroesophageal reflux (1 patient).

In the non-renal salt deplete group, 0.9% saline was utilised in 21 patients which resulted in a 7 mmol/L (5–10.5) Δ Na over the first 24 h period, 3.25 mmol/L (1–4.25) over the second 24 h period and 1 mmol/L (− 1.25–2) mmol/L change over the third 24 h period.

Hypertonic (3%) saline was prescribed in 6 patients, whose median baseline serum Na was 114.5 mmol/L (111–117). The cause of hyponatraemia was polydipsia in 3 patients, SIADH in 2 patients and was not documented in 1 patient. The median volume infused was 305 mL (300–528). The median Na post treatment was 121 mmol/L (119.3–126.5). The median Δ Na was 11 mmol/L (5–13).

Inappropriate rapid correction of serum Na with a > 10 mmol/L increase in the first 24 h occurred in 13 patients (8.6%), 11 of whom had chronic or an unknown duration of hyponatraemia. Treatment was given for over correction in 3 patients (2%). The treatment utilised was Desmopressin and intravenous 5% dextrose in 2 patients and intravenous 5% dextrose alone in 1 patient. Two patients had a >18 mmol/L increase in sodium over 48 h, 1 of which had chronic hyponatraemia. Neither of these patients received treatment for over correction.

### Outcomes

There were no cases of osmotic demyelination. The median length of stay was 8 days (4–18.5). There was no significant correlation between nadir serum Na and length of stay using Spearman’s rank order correlation, *R* = 0.003, *P* = 0.97. Mortality of the whole cohort was 11.2% (17 pts). There was a weak but statistically significant correlation between nadir serum Na and mortality (*R* = 0.18, *P* = 0.031) using logistic regression modelling.

## Discussion

This study of hospitalized inpatients with moderate to severe hyponatraemia is the first to examine the investigation and management of this condition in the Australian setting. Our data suggest that inpatient hyponatraemia is often inadequately investigated, leading to errors in diagnosis and treatment inconsistencies.

Investigation of hyponatraemia was often incomplete, with less than half of the cohort having urine Na and osmolality performed. Similarly, thyroid function tests and morning cortisol levels were often omitted in euvolaemic patients. Comparable data have been reported by other studies conducted internationally, with adequate investigation of hyponatraemia occurring in only 26–47% of patients [16–18]. Likewise, Huda et al., found only 27% of patients had a urine osmolality and only 10% had urine sodium performed [16].

In this cohort, euvolaemic hyponatraemia was most frequently described. Other studies have found that SIADH was the most common cause of hyponatraemia [3, 16] but following adjudication by the investigators, only 21.1% were judged likely to have SIADH. As 31.6% of patients did not have a diagnosis documented and 19.7% of patients had inadequate investigation to retrospectively determine a cause of hyponatraemia, it is likely that many undiagnosed patients had SIADH to account for this discrepancy. Diagnostic precision was also examined by Huda et al., who found the inpatient diagnosis was inconsistent with the available clinical and investigative data in 42% [16].

Within the SIADH cohort, fluid restriction was first line treatment in the majority of patients, however the degree of fluid restriction appeared to be arbitrary. Urine output for the preceding 24 h was not readily available. In no case was a calculated urine/plasma electrolyte ratio utilised to determine adequate fluid restriction volumes. Patients with a urine osmolality greater or less than 400 mOsm/kg did not have a significant different Δ Na over 3 days of fluid restriction, which may be due to the small number of patients analysed. Other groups have demonstrated that a higher urine osmolality correlated with non-response to fluid restriction [11]. Winzeler et al. prospectively analysed 82 patients with SIADH and treated with fluid restriction of <1000 mL. They found a higher median urine osmolality of 432 mOsm/kg (IQR 331–597) in non-responders compared with 385 mOsm/kg (IQR 301–438) in responders (*P* = 0.03) [11].

Second line treatments were only used in a small proportion of patients. Of the patients who received urea there was a substantial increase in serum Na levels over the following 72 h, noting that this group had either already failed fluid restriction or were unable to be fluid restricted. Adverse effects, while common, were mild and easily tolerable. These pilot data resulted in a change of practice at our institution, such that urea is now routinely used in patients with SIADH who do not respond to fluid restriction within 24-48 h.

Previous research which supports the use of urea for the management of SIADH includes a retrospective analysis of 42 patients who were admitted to the Intensive care unit [19]. Patients were treated with a standardised protocol which implemented urea after clinical deterioration (measured as drop in GCS by 2 points) or serum Na <130 mmol/L despite 0.9% saline. Hyponatraemia was corrected in all patients and was well tolerated with no adverse effects [19]. Similar data were obtained by Decaux et al. who compared 50 patients with mild hyponatraemia to 35 patients with severe hyponatraemia treated with urea. All the patients in the mild and severe group achieved resolution of hyponatraemia, however 6 patients in the mild group developed hypernatraemia without any associated adverse outcomes [20]. The only prospective data to date compared 1 year of treatment with a vaptan followed by 1 year of treatment with urea in 13 patients. In this cohort, urea was shown to have the same efficacy as vaptans [21].

Hypovolaemic patients treated with 0.9% saline demonstrated a significant increase in serum Na, suggesting that treatment of non-renal salt wasting is less refractory than SIADH. Hypertonic (3%) saline was utilized in cases of severe symptomatic hyponatraemia using varying volumes and over different time periods. There was a significant increase in serum Na using this mode of treatment, however the time frame in which this occurred varied between patients. Frequent monitoring of serum Na is necessary to prevent over correction.

While acknowledging the limitations of the retrospective design, this is the first Australian study to examine the investigation and management of moderate to severe inpatient hyponatraemia in detail, including the efficacy of fluid restriction and in particular the use of second line treatments. The data have been analysed according to documentation by the treating team, as well as reanalysed by the investigators. It is possible that volume status and diagnosis were discussed but not recorded in patients’ notes, however laboratory data has been captured for all analysed patients.

## Conclusion

Hyponatraemia is often inadequately investigated, which may lead to diagnostic and management errors. There is a need for improved guidance to clinicians with respect to the recommended initial fluid restriction volume. For patients who do not respond to fluid restriction within 24–48 h or who are unable to be fluid restricted, treatment with urea resulted in improvement in serum Na. Hypertonic (3%) saline remains the treatment of choice for severe symptomatic hyponatraemia but must be carefully monitored.

## Abbreviations

∆ Na: Change in serum sodium
ADH: Antidiuretic hormone
GCS: Glascow coma scale
IQR: Interquartile range
Na: Sodium
SIADH: Syndrome of inappropriate antidiuretic hormone secretion
